# Congenital Simple Hamartoma of Retinal Pigment Epithelium: Clinical and Imaging Findings

**DOI:** 10.1155/2012/654502

**Published:** 2012-04-09

**Authors:** Mehmet Yasin Teke, Pinar Ç. Özdal, Figen Batioglu, Ufuk Elgin, Faruk Öztürk

**Affiliations:** ^1^Retina Department, Ulucanlar Eye Research and Training Hospital, 06460 Ankara, Turkey; ^2^Department of Ophthalmology, Ankara University Medical School, 06460 Ankara, Turkey

## Abstract

Congenital simple hamartoma of retinal pigment epithelium (CSHRPE) is a rare, asymptomatic, and incidentally detected benign lesion. However, it is very important to do the differential diagnosis from other pigmented retinal lesions. Its clinical presentation and imaging findings are very helpful in doing this differentiation. This paper presents clinical and imaging findings of a 56-year-old woman with incidentally detected CSHRPE. The lesion was small, heavily pigmented, well circumscribed, and slightly elevated. Optical coherence tomography (OCT) scanning was diagnostic and showed an elevated retina at the site of the lesion, increased optical reflectivity on its inner surface, optical shadowing of deeper structures, and clearly cut tumor margins. Ocular ultrasonography, fluorescein angiography, and fundus autofluorescence imaging which is firstly described in this report did not show any characteristic finding.

## 1. Introduction

Simple hamartoma of retinal pigment epithelium, firstly recognized by Laqua [1] in 1981, is an uncommon entity presumed to be congenital. Since its clinical description and denomination as “retinal hamartoma” in 1989 by Gass [2]^,^ there are very few cases reported in the literature [3–7]. The largest case series has been reported by Shields et al. in 2003 and included clinical characteristics of five cases [3].

We report ophthalmoscopic and imaging findings of a case with congenital simple hamartoma of retinal pigment epithelium (CSRPEH). To the best of our knowledge, although fluorescein angiography (FA) and optical coherence tomography (OCT) characteristics had already been described [3–7], this is the first case showing fundus autofluorescence (FAF) imaging of this rare entity.

## 2. Case Report

**Figure 1:**
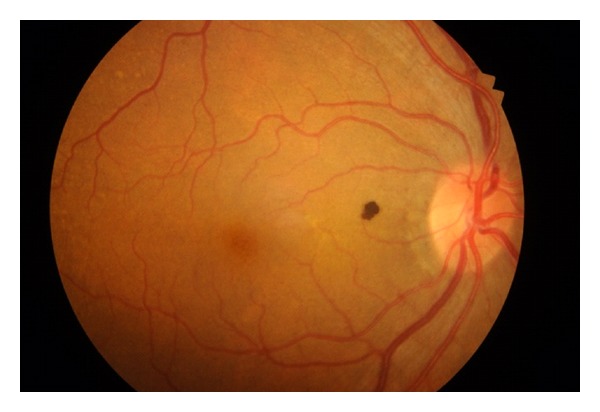
Heavily pigmented, small, well-circumscribed, and slightly elevated lesion located superonasal to the fovea and temporal to the optic disc.

**Figure 2:**
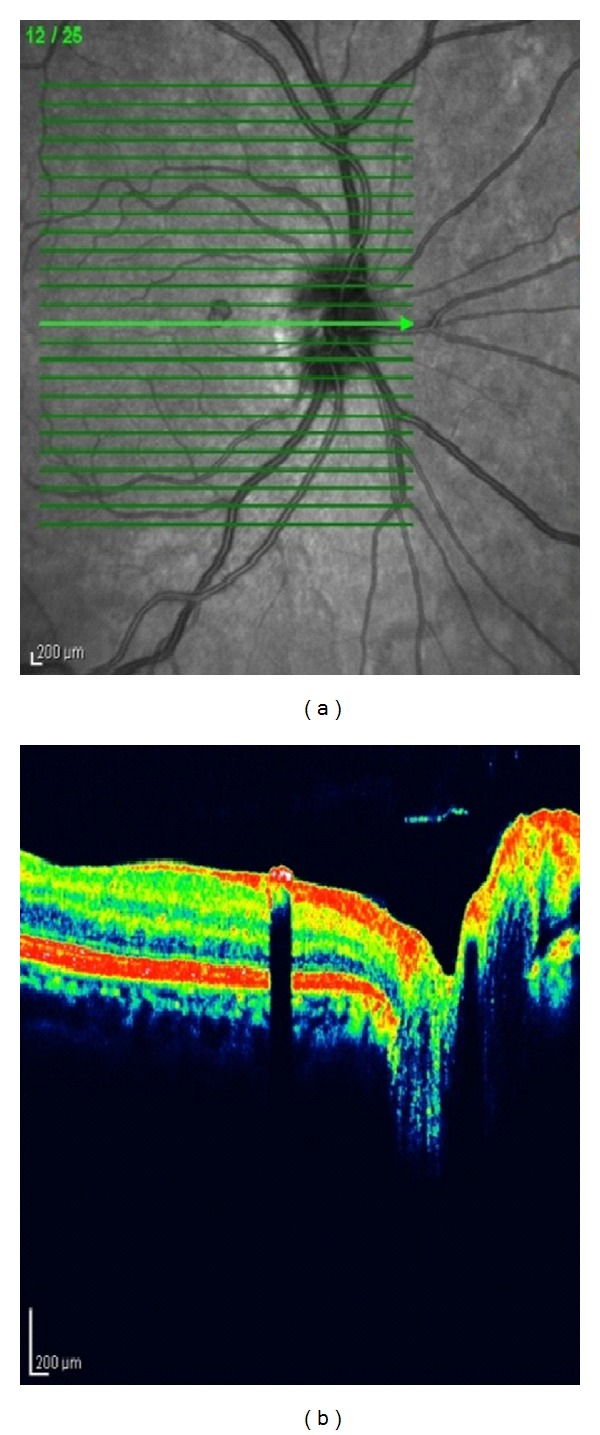
Optical coherence tomography scanning showed an elevated retina at the site of the lesion, increased optical reflectivity on its inner surface, optical shadowing of deeper structures, and clearly cut tumor margins.

**Figure 3:**
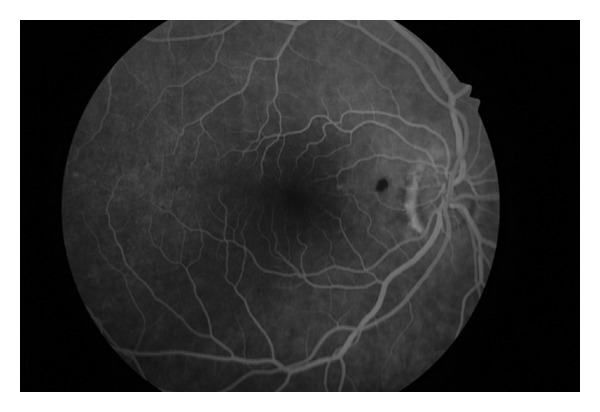
Fluorescein angiography showed blockage of fluorescence due to pigmented tumor and no leakage was observed.

**Figure 4:**
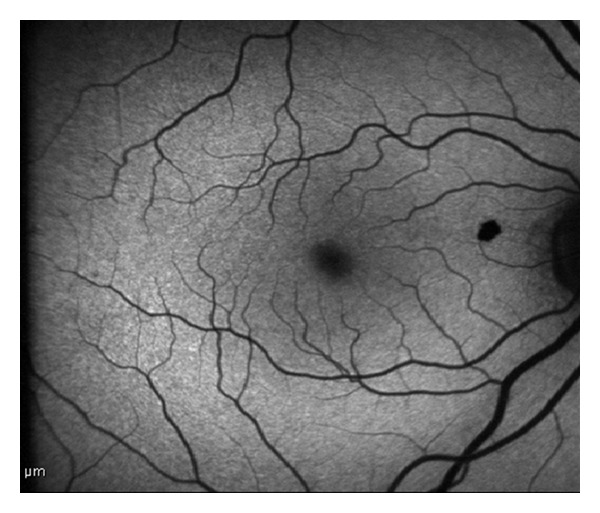
Fundus autofluorescence imaging showed obscurenesse of the background autofluorescence at the site of the lesion.

A 56-year-old woman presented for a routine eye examination had been told to have a nevus in the back of her right eye and referred to the “Retina Department” of our hospital. The patient was completely asymptomatic. The visual acuity was 20/20 in both eyes. Biomicroscopic findings and intraocular pressure were completely normal. Dilated fundus examination revealed a heavily pigmented, small, well-circumscribed, and slightly elevated lesion located superonasal to the fovea and temporal to the optic disc (Figure 1). There was no associated retinal traction, hemorrhage, or subretinal fluid. Fundus examination of the left eye showed no abnormality. Fluorescein angiography showed blockage of fluorescence due to pigmented tumor and there was no leakage (Figure 2). At OCT scanning, an elevated retina at the site of the lesion, increased optical reflectivity on its inner surface, optical shadowing of deeper structures, and clearly cut tumor margins were noted. The surrounding retina and choroid had normal structures (Figure 3). On the FAF imaging, blockage of the background autofluorescence was observed at the site of the lesion (Figure 4). An ultrasonographic evaluation did not show any abnormality and the mass could not be seen.

 Examination and imaging findings of the left eye were completely normal.

## 3. Discussion

CSRPEH is a heavily pigmented, well circumscribed and nodular benign tumor usually located adjacent to the foveola or more rarely at the foveal center. This lesion involving the full-thickness retina may be associated by retinal traction, exudation, dilated feeding and draining retinal vessels and pigmented vitreous cells [3, 8]. None of these associated findings was present in our case. This lesion is completely different than combined hamartoma of the retina and RPE. The combined hamartoma is a gray-green, sometimes non-pigmented tractional mass located usually at the optic nerve head. This full-thickness retinal mass is associated with vitreoretinal interface disturbance, prominent corkscrew vascular tortuosity and peripheral retinal vascular straightening and traction. OCT displays massive retinal thickening with cystoid spaces and irregular epiretinal membrane [3, 8].

CSRPEH is asymptomatic and detected incidentally in most of cases as in ours. Because it does not grow and remains stationary, the visual prognosis is good especially for lesions located extrafoveally [3].

Ocular ultrasonography demonstrates an echogenic and nodular mass with moderate to high internal reflectivity and without shadowing [3, 5]. Ultrasonographic evaluation of our case did not show these findings. This might be due to its very small size.

Fluorescein angiography of CSRPEH shows nonfluorescence throughout the angiogram as in our case. Late hyperfluorescence which is not observed in our case may be seen in few cases [3, 6].

OCT findings of CSRPEH were first described by Shields et al. in 2004 [4]. Then Shukla et al. reported the OCT as a useful and non-invasive adjunct for diagnosis of this rare tumor [5]. OCT scanning of our case showed an elevated retina at the site of the lesion, increased optical reflectivity on its inner surface, optical shadowing of deeper structures and clearly cut tumor margins. The surrounding retina and choroid had normal structures. These findings were consistent with those of previously reported cases [4–6]. The normal appearance of adjacent underlying choriocapillaris and choroid in OCT was considered helpful in excluding the diagnosis of choroidal nevus or melanoma [4].

Fundus autofluorescence findings of this rare lesion had not been described before and were not characteristic. Because FAF is known to originate from the lipofuscin and the content of CSHRPE is melanin, a blockage of background autofluorescence at the site of heavily pigmented lesion was observed.

Varied pigmented lesions such as congenital hypertrophy of the RPE, hyperplasia of RPE, intraretinal foreign body, adenoma and adenocarcinoma of the RPE, combined hamartoma of retina and RPE should be considered in the differential diagnosis of CSRPEH [3–6]. Clinical features such as the visual disturbance, location, color, associated retinal, and vascular involvement and imaging findings are very helpful in making the distinction between these lesions.
